# Associations between subcutaneous adipocyte hypertrophy and nonalcoholic fatty liver disease

**DOI:** 10.1038/s41598-022-24482-1

**Published:** 2022-11-28

**Authors:** Magnus Holmer, Hannes Hagström, Ping Chen, Olof Danielsson, Myriam Aouadi, Mikael Rydén, Per Stål

**Affiliations:** 1 Division of Liver and Pancreatic Diseases, Department of Upper GI, Huddinge C1-77, Karolinska University Hospital, 141 86 Stockholm, Sweden; 2 Division of Gastroenterology, Department of Medicine, Huddinge, Karolinska Institutet, Stockholm, Sweden; 3 Division of Clinical Epidemiology, Department of Medicine, Solna, Karolinska Institutet, Stockholm, Sweden; 4 Department of Medicine, Huddinge, Center for Infectious Medicine (CIM), Karolinska Institutet, Karolinska University Hospital, Stockholm, Sweden; 5 Division of Pathology, Department of Laboratory Medicine, Huddinge, Karolinska Institutet, Karolinska University Hospital, Stockholm, Sweden; 6 Endocrinology Unit, Department of Medicine, Huddinge, Karolinska Institutet, Karolinska University Hospital, Stockholm, Sweden

**Keywords:** Non-alcoholic fatty liver disease, Non-alcoholic steatohepatitis, Diagnostic markers, Predictive markers, Metabolic syndrome

## Abstract

Adipocyte hypertrophy and expression of adipokines in subcutaneous adipose tissue (SAT) have been linked to steatosis, nonalcoholic steatohepatitis (NASH) and fibrosis in morbidly obese (BMI ≥ 40 kg/m^2^) subjects. It is unknown if this is also true for subjects with NAFLD with lesser degrees of obesity (BMI < 35 kg/m^2^). Thirty-two subjects with biopsy-proven NAFLD and 15 non-diabetic controls matched for BMI underwent fine-needle biopsies of SAT. Adipocyte volume was calculated. RNA-sequencing of SAT was performed in a subset of 20 NAFLD patients. Adipocyte volume and gene expression levels were correlated to the presence of NASH or significant fibrosis. Subjects with NAFLD had larger adipocyte volume compared with controls, (1939 pL, 95% CI 1130–1662 vs. 854 pL, 95% CI 781–926, *p* < 0.001). There was no association between adipocyte volume and the presence of NASH. Gene expression of adipokines previously described to correlate with NASH in morbid obesity, was not associated with NASH or fibrosis. Our results suggest that persons with NAFLD have larger SAT adipocytes compared with controls and that adipocytes are involved in the pathophysiology of hepatic steatosis in NAFLD. However, adipocyte volume was not associated with NASH or fibrosis in NAFLD subjects with varying degrees of obesity.

## Introduction

The prevalence of nonalcoholic fatty liver disease (NAFLD) is increasing globally and is today estimated to be approximately 25%^1^. This development parallels the increase of obesity and type 2 diabetes (T2D)^2^. NAFLD and T2D share insulin resistance (IR) as a common pathophysiological hallmark^3,4^. Recent data from the US, Europe, Australia, and New Zeeland shows that NAFLD is the most rapidly increasing indication for liver transplantation^5–7^. However, the majority of NAFLD patients will not develop advanced liver disease^8^. A major challenge is to identify the minority of NAFLD patients who carries a risk of disease progression^9^. Although the presence of liver fibrosis is the most important prognostic factor for subsequent morbidity and mortality in liver disease, nonalcoholic steatohepatitis (NASH) has been identified as an important predictor of disease progression^10–12^. The underlying pathogenic mechanisms as to why a subset of patients develop NASH is largely unknown. One hypothesis would be an interplay between the abdominal subcutaneous adipose tissue (SAT) and the liver through the release of adipokines^13^. Both morphological and physiological characteristics of SAT have been associated to NAFLD development and disease progression^14,15^. It has been demonstrated that adipocyte size correlates to the level of steatosis partly independent of other traits of the metabolic syndrome^16^. Further, in studies of morbidly obese patients undergoing bariatric surgery, adipocyte hypertrophy of SAT was associated with a higher prevalence of NAFLD, NASH and hepatic fibrosis^17,18^. However, it is unknown if this association applies also to patients with NAFLD with a lower body mass index (BMI).

Through the secretion of pro- or anti-inflammatory adipokines, the adipose tissue can exert direct effects on the liver^19–21^. Previous studies have found that the expression of specific adipokines correlate both to the presence of NASH and to hepatic fibrosis in NAFLD^22–25^. Despite several promising candidates, no single marker has been identified that alone can predict the presence of NASH or hepatic fibrosis with a high sensitivity. More recent studies have used gene array techniques to identify panels of adipokines that are associated with NASH and hepatic fibrosis^26,27^. These studies are based on selected cohorts of patients with morbid obesity (BMI ≥ 40) who are undergoing bariatric surgery. It is not known whether these patterns of adipokine expressions can predict presence of NASH or significant hepatic fibrosis in an unselected NAFLD population.

The aim of this study was to investigate the association between adipocyte volume or expression of adipokines in SAT, with features of NASH or hepatic fibrosis in patients with biopsy proven NAFLD.

## Results

### Cohort characteristics

In total, 32 patients with a biopsy-proven diagnosis of NAFLD were included. Fifty-three percent were male, and the mean age was 55.2 years. In total, 20 (62.5%) subjects had NASH and 18 (56%) had significant or advanced fibrosis (F2-4). Fifteen subjects (47%) had T2D. The prevalence of T2D was higher among subjects with NASH, n = 13 (65%), compared to subjects without NASH, n = 2 (17%), (*p* = 0.008). HOMA-IR (8.2 vs. 4.7, *p* = 0.008) and BMI (31.8 vs. 27.9 kg/m^2^, *p* = 0.019) was higher in the NASH group compared to non-NASH. Characteristics of the study cohort at the time of SAT biopsy are shown in Table 1.

**Table 1:** Cohort characteristics.

	All cases	Non-NASH	NASH	*P*	Controls
	n = 32	n = 12	n = 20		n = 15
Sex, male, n (%)	17 (53.1)	8 (66.7)	9 (45)	0.234	5 (33.3)
Age, years, mean (± SD)	55.2 (14.4)	52.9 (16.5)	56.5 (13.1)	0.503	55.7 (10.3)
BMI, kg/m^2^, mean (± SD)	30.3 (4.6)	27.9 (1.5)	31.7 (5.3)	**0.019**	26.2 (2.4)
T2D (%)	15 (46.9)	2 (16.7)	13 (65.0)	**0.008**	0 (0)
HOMA-IR, median (IQR)	7.1 (5.1–10.9)	4.7 (4.0–6.7)	8.2 (6.9–13.2)	**0.008**	NA
ALT U/L, mean (± SD)	87.8 (51.5)	75.6 (42.5)	90.5 (54.1)	0.446	NA
**Fibrosis stage**					
No fibrosis, n (%)	3 (9)	1 (10)	2 (9)		
Stage 1, n (%)	11 (34)	6 (60)	5 (24)		
Stage 2, n (%)	10 (31)	3 (30)	7 (33)		
Stage 3, n (%)	6 (19)	0 (0)	6 (29)		
Stage 4, n (%)	2 (6)	0 (0)	2 (9)		
**Steatosis**					
Score 1, n (%)	3 (9.4)	2 (16.6)	1 (5.0)		
Score 2, n (%)	11 (34.4)	5 (41.7)	6 (30.0)		
Score 3, n (%)	18 (56.3)	5 (41.7)	13 (65.0)		
**Lobular inflammation**					
Grade 0, n (%)	5 (15.6)	5 (41.7)	0 (0)		
Grade 1, n (%)	21 (65.6)	7 (58.3)	14 (70.0)		
Grade 2, n (%)	5 (15.6)	0 (0)	5 (25.0)		
Grade 3, n (%)	1 (3.2)	0 (0)	1 (5.0)		
**Hepatocyte ballooning**					
Grade 0, n (%)	11 (34.4)	11 (91.7)	0 (0)		
Grade 1, n (%)	13 (40.6)	1 (8.3)	12 (60.0)		
Grade 2, n (%)	8 (25.0)	0 (0)	8 (40.0)		

BMI, Body Mass Index; T2D, Type 2 Diabetes; HOMA-IR, Homeostatic Model Assessment for Insulin Resistance; ALT, Alanine aminotransferase; NASH, Nonalcoholic Steatohepatitis; NA, data not available.

Values in bold denotes significance at the level of *p* < 0.05.

*P* denotes difference between the subgroups non-NASH and NASH.

### Adipocyte morphology and different traits of NAFLD

Subjects with NAFLD had significantly larger adipocytes compared to controls matched for BMI in both unadjusted analysis (*p* < 0.001) and when adjusting for sex, BMI and T2D (*p* < 0.001) (Table 2A.). In the sensitivity analysis, after excluding subjects with T2D from the NAFLD group, the difference remained significant (1855 pL, 95% CI 1559–2151 in non-diabetic NAFLD vs. 854 pL 95% CI 781–926 in controls; *p* < 0.001).

**Table 2:** Mean cell volume of adipocytes in A) 15 metabolically healthy controls matched on body mass index versus 15 subjects with NAFLD, B) NAFLD subjects without NASH versus subjects with NASH and, C) NAFLD subjects with low (F0-1) versus high (F2-4) stage of fibrosis.

**A**				
Cell volume controls, n = 15 pL, mean (± SD)	Cell volume NAFLD, n = 15, pL, mean (± SD)	Delta cell volume (95%CI)	*p*	p*
854 (131)	1939 (633)	1085 (744–1427)	< 0.001	< 0.001
**B**				
Cell volume non-NASH n = 12 pL, mean (± SD)	Cell volume NASH, n = 20 pL, mean (± SD)	Delta cell volume (95%CI)	*p*	p*
1745 (573)	2205 (641)	460 (− 4 to 925)	0.052	0.126
**C**				
Cell volume F0-1, n = 14 pL, mean (± SD)	Cell volume F2-4, n = 18 pL, mean (± SD)	Delta cell volume (95%CI)	*p*	p*
1940 (748)	2099 (564)	159 (− 323 to 641)	0.504	0.99

P* denotes significance for multiple linear regression adjusting for sex, body mass index and type 2 diabetes mellitus.

*P* denotes significance for simple linear regression analysis.

**Figure 1:**
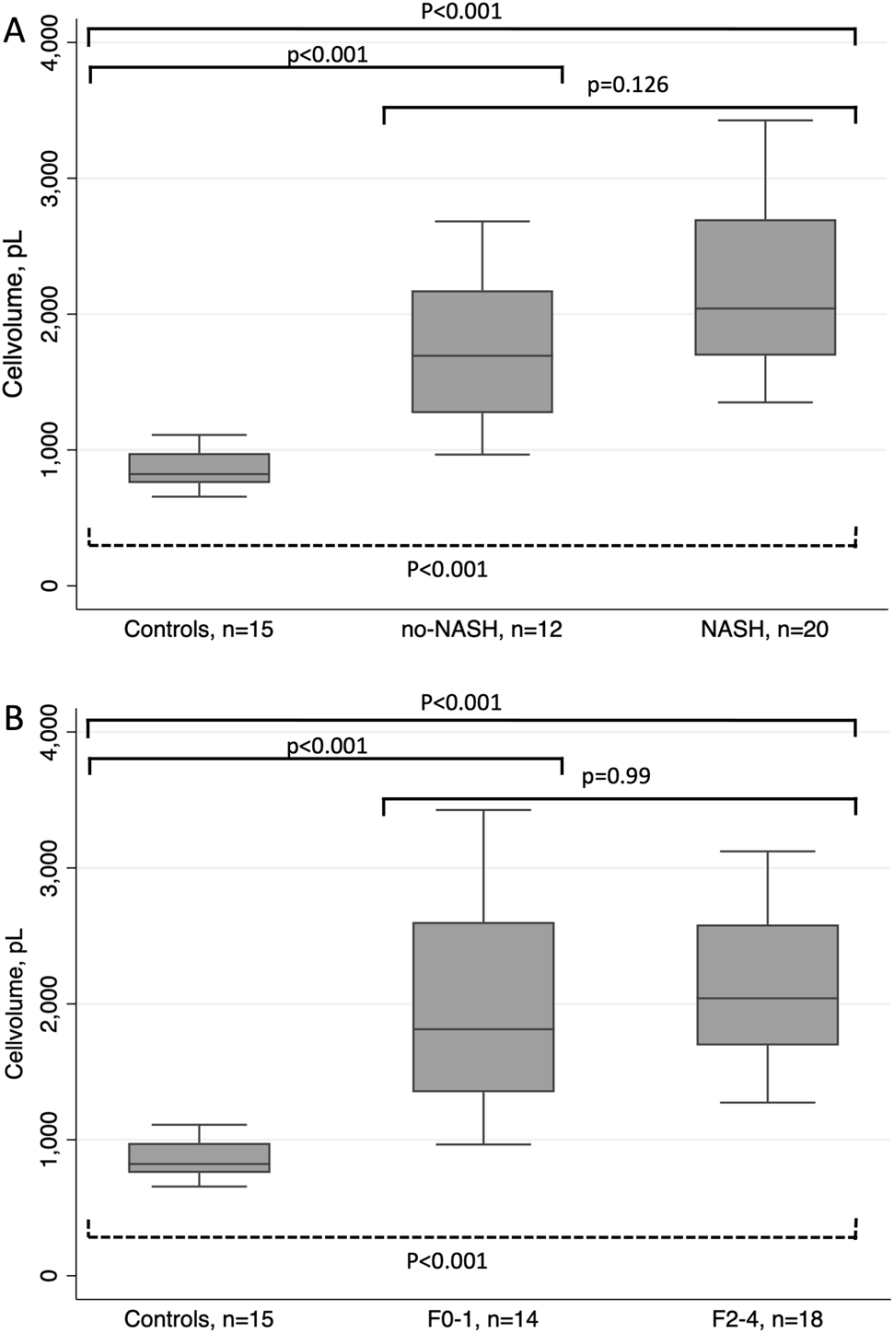
Boxplot showing comparison of adipocyte volume between healthy controls matched for BMI and (**A**) NASH and non-NASH and (**B**) no- or mild fibrosis (F0-1) versus significant or advanced fibrosis (F2-4). P at solid brackets denotes significance for multiple regression analysis adjusted for T2D, sex and BMI. P at dashed bracket denotes significance for *Kruskal–Wallis test*.

There was a trend towards a significant difference in adipocyte volume between subjects with NASH and non-NASH in the simple regression analysis, but not in the multiple regression adjusting for BMI, sex and T2D (Table 2B and Fig. 1A). No difference in adipocyte volume was detected between subjects with no or low-stage fibrosis compared with those with significant or advanced fibrosis (Table 2C and Fig. 1B). No association was observed between adipocyte volume and HOMA-IR within the NAFLD cohort.

### Gene expression of adipokines in SAT and prediction of NASH and fibrosis

**Figure 2:**
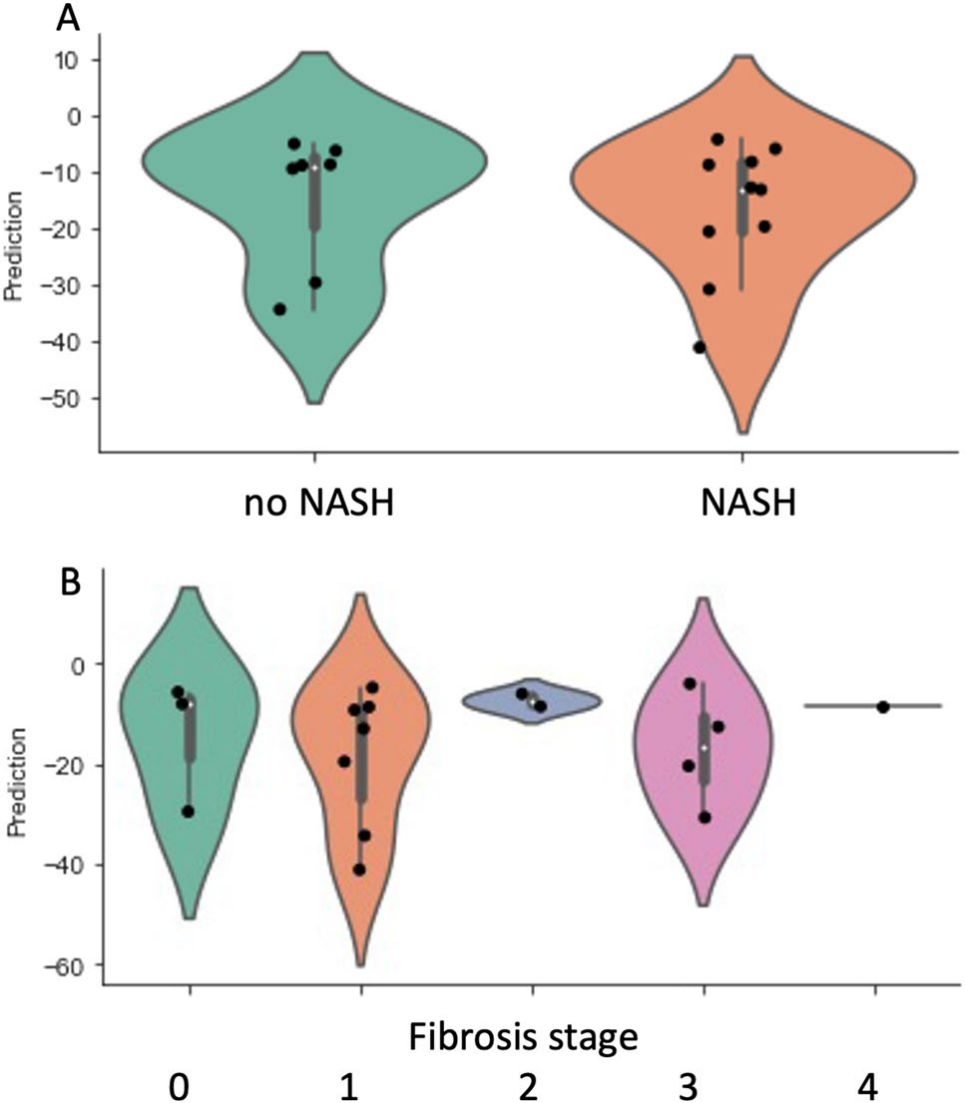
Violin plot showing the correlation between a previously published gene expression model and (**A**) NASH versus non-NASH, and (**B**) liver fibrosis stage. The y-axis (prediction) shows values calculated as the output of a published statistical model based on the expression of five genes in subcutaneous adipose tissue (CCL2, DMRT2, GADD45B, IL1RN, and IL8) ^26^.

Applying the previously published 5-gene signature (CCL2, DMRT2, GADD45B, IL1RN, and IL8)^26^ to our cohort, we found that this model was not able to accurately predict NASH or fibrosis stage (Fig. 2). Next, we evaluated the five genes separately in a multiple logistic regression analysis adjusting for age, sex, BMI and T2D, but no association was found between the expression of either of the genes and presence of NASH or fibrosis stage (data not shown).

We also explored our gene expression data from SAT for genes encoding adipokines and proinflammatory cytokines associated with NASH and fibrosis: Adiponectin, Leptin, TNF-α, IL-6, and IL-8. However, none of these adipokines were detected as differentially expressed genes between NASH and non-NASH or fibrosis F0-1 and F2-4 in the RNA-sequencing.

### Genes differently expressed in subjects with NASH

**Figure 3:**
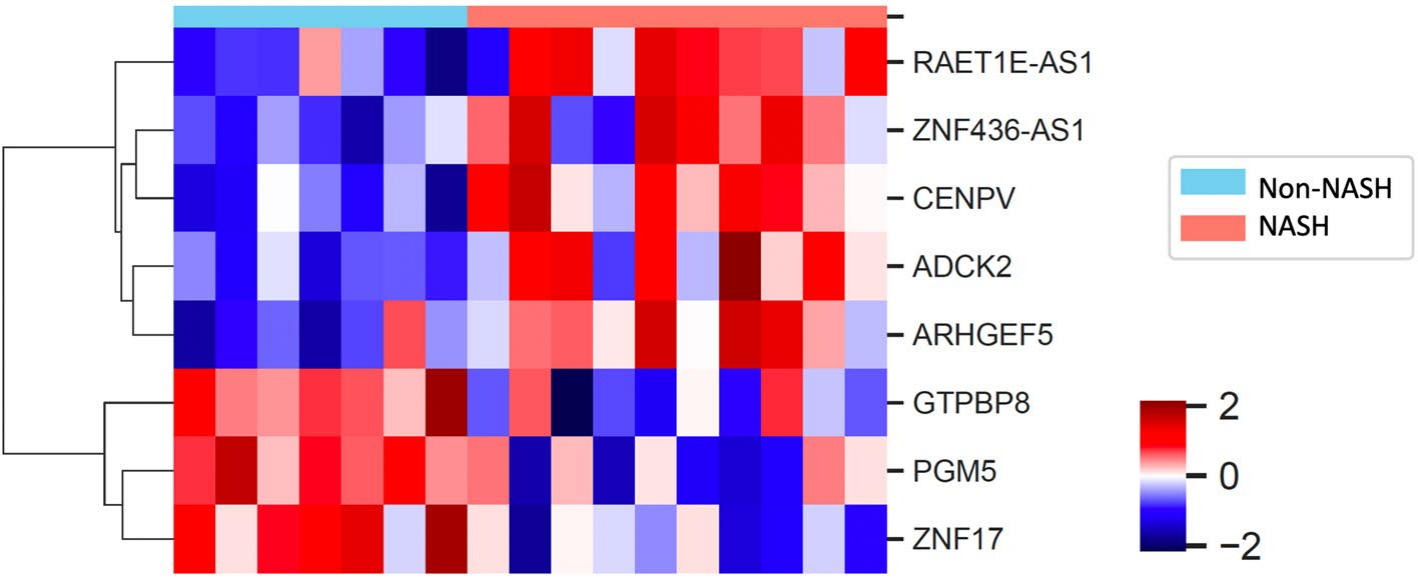
Heatmap showing expression of the 8 genes differently expressed in NASH compared to non-NASH based on Z scores.

Since we found no association between previously described adipokines and presence of NASH or fibrosis, we went on to do an exploratory investigation of the RNA-sequencing data. In total, 12,171 genes were analyzed and 86 showed significant association with NASH status from linear regression analysis. We further performed differential expression analysis on these 86 genes by comparing their expression in NASH subjects versus non-NASH subjects, leading to 31 genes with ANOVA *p*-value < 0.05. After Benjamini–Hochberg multiple hypothesis correction, 8 genes reached adjusted *p*-value < 0.05. For a more detailed description of all DEGs in the RNA-sequencing analysis, see Fig. 3 and Supplementary Table.

## Discussion

In this cross-sectional study, we evaluated if adipocyte hypertrophy in subcutaneous adipose tissue was more common in subjects with NAFLD or associated with the presence of NASH. It is expected to find adipocyte hypertrophy in obese patients and consequently, in those with an obesity-related disease such as NAFLD^28^. However, the degree of obesity at which NAFLD occurs varies significantly between individuals^29,30^. In the present study, all subjects with NAFLD had hypertrophic adipocytes, independently of BMI and the presence of T2D compared with healthy controls. This result is in part congruent with the finding of a Finnish study in which subcutaneous adipose morphology from 119 non-diabetic subjects was correlated to the degree of liver steatosis measured with Magnetic Resonance Spectroscopy. They showed that 21% of the variation in liver fat content was explained by adipocyte size alone^16^. To our knowledge, our study is the first to confirm these findings in a cohort with biopsy proven NAFLD, which also includes subjects with T2D.

The results of the present study suggest that adipocyte hypertrophy is a common morphologic hallmark of SAT present in NAFLD and of importance for the pathophysiology of the disease. Previous studies have shown that with increasing levels of obesity and IR, hypertrophic adipocytes eventually reach a critical volume^31,32^. At this stage, lipolysis leads to an increased outflow of FFA to the circulation which can cause deposition of ectopic fat in the liver^32–34^. However, the BMI threshold at which adipocytes reach their maximum volume differs between individuals, just as NAFLD can occur at different degrees of overweight. From our results, we hypothesize that individuals who are inclined to develop adipocyte hypertrophy have a predisposition to develop NAFLD as a part of their lipid dysmetabolism.

We found no association between adipocyte volume in SAT and the presence of NASH or significant or advanced fibrosis, although there was a trend towards a significant difference in adipocyte volume associated with NASH. Our results comply with those of an Australian study on 216 obese subjects undergoing bariatric surgery, with a mean BMI of 46 kg/m^2^. They found no significant correlation between adipocyte diameter in SAT and histologically defined NASH or fibrosis^35^. However, in a similar study, adipocyte volume correlated positively to the presence of NASH, but only in females^18^. They collected histological samples from SAT and liver biopsies from 668 obese patients who underwent bariatric surgery. The population in that study was composed of morbidly obese subjects with a baseline BMI of 47.5 kg/m^2^ and a high proportion of females (78%). The lack of an association between SAT morphology and NASH or fibrosis suggests that the mechanisms behind liver inflammation and fibrosis is more complex than that of simple steatosis.

Next, we explored the expressions of specific adipokines in SAT and their association with NASH and significant fibrosis. First, we applied the model first described by du Plessis et al.^26^. We could not replicate any correlation between the previously published score and the presence of NASH or significant fibrosis. Like several other studies investigating the link between adipose tissue and NAFLD, the original trial evaluated subjects undergoing bariatric surgery. The median BMI was 40–42 kg/m^2^ and the proportion of females was approximately 75%^19,27^. It should be acknowledged that these studies were meticulously performed and based on large materials. Further, they used validation cohorts to strengthen their findings. However, the characteristics of the study cohorts might not be representative of the typical NAFLD population monitored in an outpatient hepatology clinic. In our opinion, the results from studies on patients with liver steatosis combined with morbid obesity, as in those undergoing bariatric surgery, can be difficult to implement in an ordinary clinical practice. The present results further strengthen that position.

Our study has several strengths. The outcome measurement of NAFLD, NASH and liver fibrosis was confirmed with liver biopsy, the gold standard for characterizing liver histopathology^36^. The biopsies were blinded and reviewed by an experienced liver pathologist. We included a group of healthy controls matched for age and BMI to compare adipocyte morphology with NAFLD patients. We performed highly accurate determination of gene expression in SAT by using RNA sequencing^37,38^.

Several weaknesses with our study should be acknowledged. The study cohort includes only 32 subjects, and the RNA-sequencing was performed in a subset of 20 subjects. Since this was an explorative cross-sectional study without an *á priori* hypothesis on the gene expression outcome, we were not able to do a calculation of statistical power. Therefore, a type II error regarding small differences in gene expressions between subgroups cannot be excluded. Likewise, our finding of a few genes associated with NASH should be interpreted with caution. The method for measuring adipocyte volume is user dependent. To address this, all samples were measured and reviewed by a single investigator (MH) and then checked for accuracy by an independent collaborator blinded to the clinical outcome. The measurements of absolute cell volume cannot be directly compared to similar analysis from other groups due to possible differences in the handling of biopsies and fixation of histopathology slides.

## Conclusion

Our findings establish an association between subcutaneous adipocyte hypertrophy and NAFLD, but not to NASH. It supports the theory that adipocyte hypertrophy is an important factor in the pathophysiology of NAFLD. Prediction models of SAT gene expression seen in morbidly obese subjects undergoing bariatric surgery cannot be translated into a typical cohort of NAFLD patients. Our finding demands for further research focusing on the interplay between NAFLD and gene expressions of subcutaneous adipose tissue in large cohorts of patients recruited from clinical settings.

## Material and methods

### Study population

Between May 2016 and April 2018 patients with biopsy proven NAFLD who were followed at the outpatient clinic at the Department of Upper GI, Karolinska University Hospital, were screened for study participation. Exclusion criteria were: (1) alcohol consumption ≤ 140 g/week for women or ≤ 210 g/week for men, (2) treatment with either insulin, Glucagon-like peptide 1-analogue or glitazones, (3) chronic liver disease other than NAFLD, (4) pregnancy or lactation, (5) coagulopathy or treatment with anti-coagulants, and (6) reduction of BMI ≥ 1,5 kg/m^2^ since time of liver biopsy. All participants signed an informed consent, and the study was approved by the Regional Ethics Committee in Stockholm, Sweden (Dnr 2011/13–31/1). All interventions were made in accordance with local regulations at Karolinska University hospital regarding clinical research and with the declaration of Helsinki. At the visit for the SAT biopsy, data on age, sex, body mass index (BMI), presence of T2D, homeostatic model assessment for insulin resistance (HOMA-IR) and alanine aminotransferase (ALT) were collected. For comparison of SAT adipocyte size, a group of 15 healthy controls were selected from a cohort of non-diabetic subjects that has been described in a previous study^39^ and were matched on BMI (to the nearest value within  +/− 1.0 kg/m^2^) in a 1:1 ratio to 15 of the NAFLD subjects. In the original study, the controls were not screened for NAFLD, but were non-diabetic (mean fP-insulin = 6.0 ± 3.3 mU/l), non-obese and with normal measures of body composition (mean waist-to-hip ratio = 0.88 ± 0.05 and mean body fat = 22 ± 8 kg). These characteristics of metabolically healthy individuals indicate a very low likelihood of them having NAFLD. Baseline characteristics of the 15 controls included in the present study are reported in Table 1.

### Liver biopsies and histological assessment

All participants had previously been diagnosed with NAFLD through percutaneous liver biopsy as part of a clinical work-up within 12 months from inclusion. All biopsies were reassessed by an expert liver pathologist (OD) blinded to clinical data. The NAFLD activity score (NAS) was calculated as the unweighted sum of the degree of steatosis (0–3), lobular inflammation (0–3) and hepatocellular ballooning (0–2) according to Kleiner et al.^40^. NASH was diagnosed according to the fatty liver inhibition of progression (FLIP) algorithm, as the presence of both fat, lobular inflammation, and ballooning^41^. Fibrosis stage was assessed according to the classification by Kleiner^40^.

### Subcutaneous adipose tissue biopsies and fat cell measures

Participants were examined after an overnight fast. Approximately 2 g of SAT was aspirated under sterile conditions using a 14 G puncture needle and a 10 ml Hepafix® aspiration syringe (Braun, Kornberg, Germany). SAT was placed on a nylon filter net (Sefar™, Heiden, Switzerland) and washed with 0.9% sterile saline solution. About 500 mg of SAT was snap frozen in liquid nitrogen and stored in − 80 °C freezer for later RNA-sequencing. The remaining SAT was fixed in formalin and embedded in paraffin. Paraffin-embedded SAT was cut in Sects. 7–10 µm thick, mounted on microscopy slides, and stained with hematoxylin & eosin using standard protocols. The CellInsight™CX5 system (Thermo Fisher Scientific, Waltham, US) was used to generate images of histological sections. Mean adipocyte diameter was measured using ImageJ software with the Watershed adipocyte segmentation plugin. Adipocyte volume was calculated by the formula described by Goldrick^42^. The rationale for this formula has been described previously^31,43^. In brief, the diameter of a sphere is calculated by the formula $\pi \times \frac{{d^{3} }}{6}$. The mean diameter of adipocytes in a histological section (*d*) is a normally distributed variable but its cube (*d*^3^) is skewed, and the arithmetic mean of *d*^3^ can therefore not be used to calculate mean fat cell volume. The average fat cell volume is instead better approximated by using the formula $\frac{\pi }{6}\left( {3\sigma^{2} \times d^{2} } \right)d$ where *σ* is the variance of the diameter.

### RNA-sequencing – measurement of adipokines in SAT

Raw reads from RNA-seq data were aligned to human genome (hg38) using the STAR aligner^44^. Uniquely aligned reads mapping to the RefSeq gene annotations were used for gene expression estimation at reads per kilobase transcript and million mapped reads (RPKMs) using the *rpkmforgenes* software^45^. Low quality samples were excluded from downstream analysis when they failed to meet the following criteria for retaining cells: (1) ≥ 50,000 sequence reads; (2) ≥ 30% of reads uniquely aligned to the genome; (3) ≥ 40% of these unique reads mapping to RefSeq annotated exons; (4) ≥ 1000 genes with RPKM ≥ 2. From the results of the RNA-sequencing we first evaluated the expression of a 5-gene signature that previously has been developed to predict the prevalence of NASH and fibrosis in morbidly obese subjects^26^. These five genes were CCL2, DMRT2, GADD45B, IL1RN, and IL8. Next, we explored other NASH-associated genes by ordinary least squares regression model using NASH status, age, sex, BMI, and DM2 as factors in combination with differential expression analysis.

### Statistics

Adipocyte volume is presented as median and interquartile range due to a skewed distribution. Continuous data are presented as mean ± SD and categorical data as percentages. For continuous parameters, differences between groups were calculated using a t-test for normally distributed data. Differences in categorical parameters were calculated using the Chi^2^-test. The Kruskal Wallis-test was used for differences between multiple groups.

For comparison of adipocyte volume, subjects with NAFLD were categorized into groups based on the presence of NASH (NASH vs. non-NASH) and fibrosis stage (non- or low stage fibrosis (F0-1) vs. significant or severe fibrosis (F2-4)). Difference in adipocyte volume was compared using a *Mann–Whitney U*-test, due to a skewed distribution of data, and multiple regression was used to adjust for age, sex, BMI and T2D. Also, as a sensitivity analysis, subjects with T2D were excluded from the NAFLD group.

We performed ordinary least squares linear regression analysis on gene expressions. Genes associated with NASH status at a significance level of *p* < 0.05 were subjected to differential expression analysis. Finally, by comparing NASH subjects and non-NASH subjects using ANOVA followed by Benjamini–Hochberg multiple hypothesis corrections, genes were identified as NASH-associated genes based on adjusted *p*-value < 0.05 and at least one condition with median log2-RPKM of 1.

## Supplementary Information

Supplementary Information.

## Data Availability

The data supporting the findings of this trial was generated at Karolinska Institutet, Stockholm, Sweden. The raw RNA-seq data has been deposited to The European Genome-phenome Archive (EGA) (https://ega-archive.org/) and is awaiting approval. The accession number is pending but will be available upon request when the data has been published. Anonymized clinical data are available from the corresponding author on reasonable request. This excludes sharing of data that conflicts with the statement of confidentiality in the informed consent.
