# Supplementary material for: Associations between subcutaneous adipocyte hypertrophy and nonalcoholic fatty liver disease
Source: Sci Rep. 2022 Nov 28;12:20519. doi: 10.1038/s41598-022-24482-1 (PMC9705525; doi:10.1038/s41598-022-24482-1)
Supplement: Supplementary file 1 — Supplementary Information. [file 41598_2022_24482_MOESM1_ESM.docx]

**Supplementary Table.** Result from analysis of differently expressed genes between non-NASH subjects compared to those with NASH. P* denotes significance for least squares regression model using NASH status, age, sex, BMI, and DM2 as factors. P** denotes differential expression analysis of non-NASH subjects versus NASH subjects using ANOVA. P*** denotes significance for Benjamini-Hochberg multiple hypothesis correction. Abbreviations: NASH= nonalcoholic steatohepatitis, BMI= body mass index, T2D= type 2 diabetes mellitus.

| GeneName | Differential expression | | P* | | | | | P** | P*** |
| --- | --- | --- | --- | --- | --- | --- | --- | --- | --- |
|  | **non-NASH** | **NASH** | **NASH** | **Age** | **Sex** | **BMI** | **T2D** |  |  |
| CENPV | 2,385 | 3,093 | **0,010** | 0,789 | 0,567 | 0,787 | 0,274 | **0,000** | **0,019** |
| ZNF17 | 1,925 | 1,674 | **0,007** | 0,711 | 0,418 | 0,991 | 0,536 | **0,001** | **0,029** |
| ARHGEF5 | 1,645 | 1,893 | **0,025** | 0,517 | 0,343 | 0,612 | 0,644 | **0,002** | **0,039** |
| RAET1E-AS1 | 0,909 | 2,322 | **0,030** | 0,480 | 0,032 | 0,457 | 0,287 | **0,003** | **0,039** |
| ADCK2 | 3,197 | 3,410 | **0,019** | 0,449 | 0,291 | 0,279 | 0,011 | **0,003** | **0,039** |
| PGM5 | 3,708 | 3,231 | **0,045** | 0,185 | 0,328 | 0,356 | 0,812 | **0,003** | **0,039** |
| GTPBP8 | 2,392 | 2,097 | **0,012** | 0,644 | 0,095 | 0,843 | 0,415 | **0,003** | **0,039** |
| ZNF436-AS1 | 3,807 | 4,319 | **0,040** | 0,986 | 0,309 | 0,858 | 0,984 | **0,004** | **0,039** |
| LINC01504 | 0,681 | 1,736 | **0,010** | 0,147 | 0,755 | 0,432 | 0,429 | **0,006** | 0,052 |
| VIT | 3,907 | 3,222 | **0,030** | 0,215 | 0,091 | 0,234 | 0,297 | **0,007** | 0,052 |
| FRMD4B | 2,716 | 2,137 | **0,012** | 0,802 | 0,079 | 0,867 | 0,360 | **0,006** | 0,052 |
| SS18L2 | 2,321 | 3,539 | **0,039** | 0,007 | 0,453 | 0,507 | 0,477 | **0,008** | 0,054 |
| CMAHP | 3,189 | 2,743 | **0,034** | 0,200 | 0,699 | 0,520 | 0,626 | **0,010** | 0,069 |
| MAMDC2 | 3,737 | 3,091 | **0,023** | 0,480 | 0,366 | 0,859 | 0,530 | **0,012** | 0,073 |
| DUS4L | 2,253 | 1,930 | **0,020** | 0,741 | 0,983 | 0,122 | 0,092 | **0,018** | 0,098 |
| P2RY14 | 3,300 | 2,752 | **0,031** | 0,880 | 0,238 | 0,562 | 0,130 | **0,018** | 0,098 |
| FBXL18 | 1,816 | 1,934 | **0,015** | 0,319 | 0,377 | 0,079 | 0,153 | **0,022** | 0,108 |
| SNORD60 | 1,786 | 0,966 | **0,036** | 0,825 | 0,723 | 0,465 | 0,613 | **0,023** | 0,108 |
| NMRK1 | 4,709 | 4,358 | **0,023** | 0,291 | 0,864 | 0,399 | 0,443 | **0,027** | 0,117 |
| CCDC121 | 1,691 | 1,537 | **0,031** | 0,932 | 0,109 | 0,496 | 0,006 | **0,027** | 0,117 |
| OARD1 | 4,651 | 4,484 | **0,046** | 0,701 | 0,437 | 0,608 | 0,174 | **0,036** | 0,124 |
| ZNF502 | 1,952 | 1,830 | **0,015** | 0,081 | 0,701 | 0,056 | 0,155 | **0,035** | 0,124 |
| ADGRG1 | 4,219 | 4,450 | **0,006** | 0,284 | 0,718 | 0,020 | 0,479 | **0,032** | 0,124 |
| ZDHHC11B | 1,659 | 1,055 | **0,027** | 0,959 | 0,975 | 0,991 | 0,087 | **0,035** | 0,124 |
| XRCC4 | 2,536 | 2,319 | **0,024** | 0,241 | 0,015 | 0,457 | 0,025 | **0,035** | 0,124 |
| FAM131A | 2,724 | 2,994 | **0,045** | 0,162 | 0,375 | 0,268 | 0,196 | **0,039** | 0,129 |
| SNORA52 | 2,537 | 2,730 | **0,022** | 0,408 | 0,277 | 0,070 | 0,143 | **0,042** | 0,131 |
| LINC01116 | 2,456 | 2,267 | **0,006** | 0,816 | 0,084 | 0,040 | 0,948 | **0,044** | 0,131 |
| ARMCX5 | 2,477 | 2,293 | **0,043** | 0,084 | 0,129 | 0,358 | 0,081 | **0,047** | 0,131 |
| DMRT3 | 1,455 | 1,188 | **0,022** | 0,549 | 0,164 | 0,950 | 0,304 | **0,047** | 0,131 |
| PHKG2 | 2,791 | 3,423 | **0,046** | 0,371 | 0,095 | 0,290 | 0,084 | **0,047** | 0,131 |
| INTS12 | 3,602 | 3,490 | **0,050** | 0,405 | 0,883 | 0,213 | 0,612 | 0,050 | 0,134 |
| RAI14 | 1,928 | 1,661 | **0,034** | 0,169 | 0,812 | 0,145 | 0,031 | 0,055 | 0,139 |
| NTPCR | 2,853 | 3,112 | **0,027** | 0,693 | 0,850 | 0,125 | 0,714 | 0,055 | 0,139 |
| SCN4A | 3,449 | 3,819 | **0,001** | 0,120 | 0,015 | 0,074 | 0,054 | 0,062 | 0,152 |
| CASTOR3 | 3,089 | 2,964 | **0,014** | 0,943 | 0,960 | 0,067 | 0,935 | 0,066 | 0,158 |
| NUP42 | 3,553 | 3,345 | **0,032** | 0,049 | 0,101 | 0,107 | 0,046 | 0,076 | 0,178 |
| HNMT | 5,722 | 5,450 | **0,046** | 0,495 | 0,061 | 0,180 | 0,071 | 0,101 | 0,222 |
| MORN1 | 1,463 | 1,695 | **0,016** | 0,010 | 0,007 | 0,104 | 0,021 | 0,101 | 0,222 |
| SCFD1 | 5,045 | 4,793 | **0,044** | 0,161 | 0,093 | 0,050 | 0,035 | 0,106 | 0,222 |
| INPP5B | 3,147 | 3,561 | **0,005** | 0,809 | 0,644 | 0,339 | 0,010 | 0,106 | 0,222 |
| BCAT1 | 2,337 | 1,976 | **0,023** | 0,354 | 0,035 | 0,254 | 0,473 | 0,112 | 0,224 |
| DZIP1L | 2,476 | 2,284 | **0,034** | 0,074 | 0,140 | 0,414 | 0,369 | 0,112 | 0,224 |
| HAT1 | 4,420 | 4,211 | **0,034** | 0,027 | 0,004 | 0,291 | 0,007 | 0,116 | 0,224 |
| DDX10 | 2,711 | 2,601 | **0,038** | 0,945 | 0,307 | 0,157 | 0,648 | 0,123 | 0,224 |
| TRIP4 | 3,862 | 3,700 | **0,009** | 0,014 | 0,002 | 0,032 | 0,066 | 0,123 | 0,224 |
| THUMPD2 | 2,459 | 2,324 | **0,030** | 0,057 | 0,075 | 0,091 | 0,466 | 0,123 | 0,224 |
| ACTR6 | 3,704 | 3,316 | **0,036** | 0,010 | 0,170 | 0,088 | 0,006 | 0,125 | 0,224 |
| PMS2P4 | 1,564 | 1,758 | **0,028** | 0,410 | 0,023 | 0,275 | 0,021 | 0,141 | 0,225 |
| LINC00653 | 2,073 | 1,784 | **0,020** | 0,127 | 0,292 | 0,009 | 0,098 | 0,140 | 0,225 |
| UCK2 | 2,293 | 2,076 | **0,022** | 0,092 | 0,601 | 0,103 | 0,294 | 0,141 | 0,225 |
| THAP9-AS1 | 4,571 | 4,386 | **0,029** | 0,065 | 0,125 | 0,063 | 0,299 | 0,132 | 0,225 |
| RSL1D1 | 4,655 | 4,580 | **0,046** | 0,830 | 0,596 | 0,964 | 0,027 | 0,128 | 0,225 |
| ABLIM2 | 1,754 | 1,513 | **0,016** | 0,526 | 0,420 | 0,215 | 0,147 | 0,135 | 0,225 |
| NPM1 | 5,847 | 5,731 | **0,041** | 0,399 | 0,996 | 0,459 | 0,195 | 0,151 | 0,237 |
| AGBL3 | 1,624 | 1,375 | **0,040** | 0,012 | 0,443 | 0,077 | 0,185 | 0,158 | 0,241 |
| CTC-338M12.4 | 2,238 | 2,462 | **0,005** | 0,243 | 0,001 | 0,541 | 0,789 | 0,160 | 0,241 |
| EHD4 | 4,404 | 4,155 | **0,040** | 0,392 | 0,128 | 0,082 | 0,921 | 0,175 | 0,258 |
| GABPB1-IT1 | 2,374 | 2,337 | **0,004** | 0,000 | 0,510 | 0,013 | 0,824 | 0,179 | 0,258 |
| ZC4H2 | 2,294 | 2,026 | **0,044** | 0,085 | 0,010 | 0,183 | 0,234 | 0,180 | 0,258 |
| APOL3 | 6,058 | 5,869 | **0,028** | 0,668 | 0,107 | 0,576 | 0,009 | 0,208 | 0,294 |
| RCL1 | 3,451 | 3,333 | **0,019** | 0,654 | 0,004 | 0,096 | 0,409 | 0,226 | 0,314 |
| GIPR | 2,233 | 2,446 | **0,017** | 0,035 | 0,483 | 0,008 | 0,467 | 0,231 | 0,315 |
| MFAP5 | 6,590 | 6,302 | **0,011** | 0,882 | 0,001 | 0,024 | 0,009 | 0,242 | 0,326 |
| ADSS1 | 3,142 | 3,531 | **0,022** | 0,066 | 0,008 | 0,177 | 0,964 | 0,253 | 0,333 |
| PMP22 | 7,585 | 7,478 | **0,044** | 0,791 | 0,327 | 0,192 | 0,514 | 0,257 | 0,333 |
| KIF1C-AS1 | 2,326 | 2,026 | **0,033** | 0,200 | 0,641 | 0,537 | 0,130 | 0,259 | 0,333 |
| NAF1 | 2,057 | 2,170 | **0,036** | 0,124 | 0,513 | 0,497 | 0,012 | 0,275 | 0,348 |
| ITPRIPL1 | 1,376 | 1,647 | **0,041** | 0,353 | 0,613 | 0,086 | 0,734 | 0,289 | 0,355 |
| TOB2 | 4,850 | 5,086 | **0,011** | 0,018 | 0,988 | 0,110 | 0,037 | 0,289 | 0,355 |
| CBWD1 | 2,638 | 2,463 | **0,044** | 0,436 | 0,672 | 0,028 | 0,370 | 0,312 | 0,378 |
| SPA17 | 1,919 | 1,781 | **0,034** | 0,646 | 0,059 | 0,025 | 0,455 | 0,327 | 0,384 |
| GOLGA3 | 3,838 | 4,037 | **0,042** | 0,230 | 0,592 | 0,777 | 0,005 | 0,330 | 0,384 |
| MARCHF3 | 1,736 | 1,984 | **0,036** | 0,800 | 0,105 | 0,092 | 0,794 | 0,321 | 0,384 |
| SLC25A25 | 3,280 | 3,476 | **0,049** | 0,409 | 0,125 | 0,036 | 0,589 | 0,462 | 0,530 |
| EIF1AY | 4,616 | 0,388 | **0,021** | 0,760 | 0,000 | 0,010 | 0,797 | 0,526 | 0,595 |
| PCNA | 4,824 | 4,839 | **0,022** | 0,073 | 0,062 | 0,003 | 0,491 | 0,578 | 0,640 |
| ZNF526 | 1,801 | 1,885 | **0,046** | 0,152 | 0,484 | 0,009 | 0,816 | 0,580 | 0,640 |
| SNORA48 | 1,280 | 1,642 | **0,027** | 0,918 | 0,988 | 0,180 | 0,062 | 0,620 | 0,675 |
| SNRPB2 | 3,781 | 3,835 | **0,041** | 0,027 | 0,221 | 0,001 | 0,386 | 0,786 | 0,845 |
| CNOT7 | 3,871 | 3,961 | **0,020** | 0,768 | 0,703 | 0,035 | 0,003 | 0,808 | 0,851 |
| INPP5A | 3,674 | 3,539 | **0,021** | 0,027 | 0,070 | 0,009 | 0,013 | 0,811 | 0,851 |
| RNF144A | 3,260 | 3,379 | **0,035** | 0,831 | 0,944 | 0,205 | 0,012 | 0,923 | 0,956 |
| SVIL | 3,572 | 3,555 | **0,035** | 0,129 | 0,040 | 0,077 | 0,099 | 0,954 | 0,977 |
| AKIP1 | 3,195 | 3,166 | **0,017** | 0,325 | 0,226 | 0,001 | 0,237 | 0,996 | 0,996 |
| SH3BP5 | 5,282 | 5,506 | **0,027** | 0,448 | 0,389 | 0,040 | 0,170 | 0,996 | 0,996 |
